# Dairy products and brain structure in French older adults

**DOI:** 10.1017/S0007114523001551

**Published:** 2024-02-14

**Authors:** Hermine Pellay, Aline Thomas, Marion Baillet, Catherine Helmer, Gwénaëlle Catheline, Corinne Marmonier, Cécilia Samieri, Catherine Féart

**Affiliations:** 1 Universty of Bordeaux, INSERM, Bordeaux Population Health, UMR1219, F-33000 Bordeaux, France; 2 CNIEL, Service Recherche Nutrition-Santé, F-75009 Paris, France; 3 Clinical and Epidemiological Research Unit, INSERM CIC1401, F-33000 Bordeaux, France; 4 Universty of Bordeaux, CNRS, INCIA, UMR5287, F-33000 Bordeaux, France; 5 Laboratoire Neuroimagerie et vie quotidienne, EPHE-PSL, F-33000 Bordeaux, France

**Keywords:** Dairy products, Brain structure, Cortical thickness, Grey matter volume, Medial temporal lobe volume, Ageing, Population-based cohort

## Abstract

Among food groups with putative benefits for brain structures, dairy products (DP) have been poorly studied. The sample included participants without dementia from the ancillary brain imaging study of the Three-City cohort who were aged 65+ years, had their DP intake assessed with a FFQ at baseline and underwent an anatomical scan 3 years (*n* 343) or 9 years (*n* 195) after completing the dietary survey. The frequencies of consumption of total DP, milk and cheese were not associated with brain structure. Compared with the lowest frequency, the highest frequency of fresh DP (F-DP) consumption (< 0·5 *v*. > 1·5 times/d) was significantly associated with a lower medial temporal lobe volume (MTLV) (*β* = −1·09 cm^3^, 95 % CI − 1·83, −0·36) 9 years later. In this population-based study of older adults, the consumption of F-DP more than 1·5 times/d was associated with a lower MTLV, which is considered an early biomarker of Alzheimer’s disease, 9 years later. This original study should be replicated in different settings before conclusions are drawn.

Dairy products (DP) are a complex food group that contains a large set of essential components that contribute to a healthy diet and potentially to better health. Indeed, DP are involved in the prevention of several age-related chronic diseases, such as type 2 diabetes^(1–4)^, protein–energy malnutrition^(5)^ and CVD^(6–8)^. In addition, several nutrients, including bioactive peptides, colostrinin, proline-rich polypeptides, *α*-lactalbumin, Ca, probiotics and B vitamins (riboflavin or B_12_), that are provided by DP have been identified as potential preventive factors of cognitive disorders due to their neuroprotective, antioxidant and/or anti-inflammatory properties^(9,10)^.

Studies focusing on the relationship between DP intakes and cognitive decline or dementia risk have shown mixed results, and the potential benefit of higher DP intakes on brain health remains debated^(11–14)^. Contradictions may partly result from differences in sociodemographic levels, dietary patterns, food and nutrient intakes, and studied DP and DP subtypes (i.e. milk, fresh DP (F-DP) and cheese). Moreover, the design of previous studies (mainly cross-sectional), the long-term insidious neurodegenerative process and the heterogeneity of the procedures used to assess cognitive performance and diagnose dementia could also partly explain the mixed results obtained to date.

As an emerging area of research in nutrition^(15)^, imaging biomarkers can help detect early Alzheimer’s disease (AD)-related changes in cognitively normal older individuals^(16)^. Investigating early markers of brain ageing through brain imaging would help to achieve a better understanding of the relationship between DP intakes and cognitive ageing over time. To our knowledge, five cross-sectional studies^(17–21)^ that investigated the association between adherence to a Mediterranean diet (MeDi) and brain structure performed additional exploratory analyses on components of the MeDi, including DP intake. Among these studies, four showed no significant association between DP consumption and imaging biomarkers^(17–20)^. However, in one study, a higher intake of total DP (T-DP), defined as a consumption higher than the sex-specific, median T-DP consumption of the study sample, was associated with higher radial diffusivity and lower fractional anisotropy values 9 years after dietary assessment, suggesting altered white matter integrity^(21)^. To our knowledge, no study has explored each DP subtype separately, while dietary, nutritional and sociodemographic characteristics may differ among milk, F-DP and cheese consumers^(22)^.

Given the potential beneficial effects of nutrients from DP on brain health and the mixed results on both dementia risk and imaging biomarkers of AD, we evaluated the associations of DP with early markers of neurodegeneration in a population-based sample of cognitively healthy French older adults. We measured the frequency of consumption of T-DP and DP subtypes by using a FFQ. We analysed three MRI markers of neurodegeneration, focusing on key regions sensitive to neuropathological processes in AD^(23–25)^. We tested whether participants who had the highest frequency consumption of T-DP and DP subtypes exhibited fewer brain structure alterations.

## Methods

### Study overview

The Three-City (3C) study is a population-based prospective cohort study conducted in three French cities (Bordeaux, Dijon and Montpellier) and was initiated in 1999–2000. This study included 9294 participants at baseline, 2104 of whom were part of the Bordeaux centre. Sociodemographic and lifestyle information, anthropometric data, neuropsychological test data and data on symptoms and medical complaints, medical history, medication use, and blood pressure were collected at home by neuropsychologists^(26)^.

In 2001–2002 (considered thereafter as the baseline of the present analysis), a comprehensive dietary survey was proposed to the participants from the Bordeaux centre; 1755 participants were included, among whom 1584 had no missing data on the main exposure variable (i.e. T-DP, milk, F-DP and cheese consumption), whose main characteristics were described above^(22)^. In addition, an ancillary brain imaging study was conducted, with two MRI exams carried out 3 years (3-year MRI exam) and 9 years (9-year MRI exam) after the completion of the dietary survey (in 2004–2006 and 2010–2011, respectively), which allowed for the estimation of cerebral volumes in 422 and 239 participants, respectively. MRI scans were offered to participants younger than 80 years of age. The exclusion criteria for the various MRI examinations were defined by the existence of the following MRI contraindications: pacemakers, issues with certain heart valves, ferromagnetic vascular clips, intraocular metallic foreign bodies, claustrophobia, major obesity and severe respiratory insufficiency.

After excluding participants with major brain pathologies (i.e. meningioma or a major cerebrovascular pathology), those with major acquisition artefacts on MRI scans and post-processing failure (seventy-eight participants at the 3-year MRI exam and thirty-eight at the 9-year MRI exam) and those with prevalent dementia at the time of the MRI exam (one participant at the 3-year MRI exam and six participants at the 9-year MRI exam), a total of 343 participants without dementia with DP intake assessed at baseline and an anatomical scan 3 years after the completion of the dietary survey, as well as 195 participants without dementia who underwent an MRI examination 9 years after the completion of the dietary survey, were included in the present study^(27)^. A flow chart is shown in online Supplementary Figure Methods.

The protocol of the 3C study was approved by the Consultative Committee for the Protection of Persons participating in Biomedical Research at Kremlin-Bicêtre University Hospital (Paris, France), and all participants provided written informed consent.

### Assessment of dairy products intake

The FFQ was administered at baseline to assess the frequency of consumption of 148 foods and beverages, divided into eleven classes (from ‘never or less than once a month’ to ‘7 times per week’), for each of the six meals/snacks per d, as previously detailed^(28)^. To test the validity and reproducibility of the FFQ, the FFQ data were compared with information from a reference method (24-h dietary recall) used in an independent subsample (*n* 105) of the 3C study. This questionnaire was validated^(28,29)^. The frequency of consumption of three DP subtypes was assessed using eight items (‘milk’, ‘natural milk or milk with cereal’, ‘coffee with milk’, ‘tea with milk’, ‘chocolate’ and ‘chicory’ for milk; ‘yogurt and cottage cheese’ for F-DP; and ‘cheese’), and their sum was used to define the T-DP frequency consumption. The daily frequency of consumption of T-DP and milk, F-DP, and cheese were categorised into three categories based on the quartile distribution of the frequency of consumption per d (low intake: first quartile; moderate intake: second and third quartiles; high intake: fourth quartile), as previously described^(22)^. In the present work, we made the connection between the frequency of consumption per d and the mean daily intake. For example, the equivalent of the highest frequency consumption of T-DP, in terms of quantity, was an average consumption of 187 mL (sd 185 mL) of milk, 123 g (sd 111 g) of F-DP and 53 g (sd 45 g) of cheese per d^(22)^.

### Assessment of brain imaging markers

The 3-year MRI exam was performed on a 1·5-T Gyroscan Interra system (Phillips Medical System), and the 9-year MRI exam was performed on a 3-T Achieva (Phillips Medical System). The acquisition protocol is described in Supplementary Methods. FreeSurfer 5.1 was used for cortical surface reconstruction and the estimation of the grey matter volume (GMV), medial temporal lobe volume (MTLV) and cortical thickness (CT) for each region of the Destrieux *et al.* parcellation atlas^(30)^.

The total GMV (in cm^3^) was calculated as the sum of the volumes of the cortical and subcortical regions, and the MTLV (in cm^3^), an early biomarker of AD^(23)^, was computed as the sum of the amygdalar, parahippocampal and hippocampal volumes of both hemispheres. The mean CT (in mm) of the cortical regions vulnerable to AD (defined as the AD signature by Dickerson *et al.* ^(25)^) was determined based on the temporal pole, parahippocampal region, inferior temporal gyrus, superior parietal lobe, precuneus/posterior cingulate cortex complex, middle and superior frontal gyrus, inferior frontal sulcus, and angular and supramarginal gyri (averaged across hemispheres). The total intracranial volume (in cm^3^) was computed as the sum of the cerebrospinal fluid, grey matter and white matter volumes.

Hence, higher volumes in these AD regions could indicate the involvement of neurodegenerative mechanisms in the preservation of brain structure^(31)^.

### Other variables

In the baseline interview, sociodemographic and lifestyle characteristics, including age, sex and education level (no education/primary school, secondary/high school, university), were recorded.

The clinical variables included four dichotomous variables: the presence of at least one ApoE4 allele (ε4 allele of apo E), a history of diabetes (self-reported diabetes or antidiabetic treatment), a history of stroke and the number of medications per d (sample median ≥ 6 medications). Stoutness was based on the Global Leadership Initiative on Malnutrition criteria^(32)^ and categorised as follows: underweight, for a BMI < 20 kg/m^2^ or < 22 kg/m² for those aged < 70 years or ≥ 70 years, respectively; normal, for a BMI of 20–27 kg/m² or 22–27 kg/m² for those aged < 70 years or ≥ 70 years, respectively; and overweight/obesity, for a BMI > 27 kg/m²^(33)^.

Dietary data included total energy intake (kcal/d) and the mean weekly frequency of consumption (times/week) of charcuterie, meat and alcohol, which are food groups often consumed by individuals with the highest frequency of cheese consumption and the lowest frequency of milk and F-DP consumption^(22,28)^.

### Statistical analyses

The SAS statistical software program (version 9.3; SAS Institute Inc.) and RStudio (version 3.6.2) were used for statistical analyses.

Linear regressions were used to estimate the multivariable associations between the frequency of consumption of T-DP, milk, F-DP and cheese at baseline (i.e. moderate and highest frequencies of consumption compared with the lowest frequency of consumption) and MRI markers (MTLV, GMV and CT in AD-vulnerable regions) shown on the 3-year MRI and 9-year MRI exams, respectively, in separated models.

To control for potential confounders, linear regressions were adjusted for age, sex, education level, ApoE4 carrier status and total intracranial volume in model 1; additionally, adjusted for diabetes, history of stroke, number of medications taken per d and stoutness in model 2; and additionally adjusted for total energy intake and the frequencies of consumption of charcuterie, meat and alcohol in model 3. For each of the three DP subtypes considered as the main exposure variable, the other two were added as covariates in all three models. Effect modifications by sex were investigated.

Missing exposure and covariate data were imputed by multiple imputations for all inferential analyses with consideration of the missing at random mechanism^(34)^. Out of the total sample who underwent the 3-year MRI exam (*n* 343), ten participants had missing data imputed for at least one confounding variable, and for those who underwent the 9-year MRI exam (*n* 195), ten participants had data imputed for at least one exposure or confounding variable (regarding the main exposure, there were two participants with missing data for milk at baseline).

Finally, a Benjamini–Hochberg correction for a false discovery rate of 0·05 was applied to avoid multiple testing bias^(35)^.

## Results

### Description of study samples

Sociodemographic characteristics, clinical variables as well as nutritional and imaging data for each sample (i.e. 3-year MRI and 9-year MRI exams) are presented in Table 1.

**Table 1.** Baseline characteristics of participants who underwent brain structure imaging 3 years (*n* 343) and 9 years (*n* 195) after the dietary survey, Bordeaux sample of the Three-City study, 2001–2002

	Participants with MRI† exam		Participants with MRI† exam	
	3 years after dietary survey (*n* 343)		9 years after dietary survey (*n* 195)	
	*n*	%	*n*	%
Sociodemographic and lifestyle characteristics				
Age (years)				
Mean	74·1		73·4	
sd	3·8		3·7	
Women	208	60·6	117	60·0
Education level				
No or primary	83	24·2	47	24·1
Secondary or high school	180	52·5	102	52·3
University	80	23·3	46	23·6
Health indicators				
ApoE4 carrier status*,‡	70	21·0	33	17·6
Diabetes	26	7·6	11	5·6
History of stroke hospitalisation	3	0·9	2	1·0
Number of medications taken/d ≥ 6	96	28·0	47	24·1
Stoutness*,§				
Underweight	34	9·9	10	5·2
Normal	172	50·3	105	54·1
Overweight/obesity	136	39·8	79	40·7
Nutritional data				
Total energy intake (kcal/d)				
Mean	1756		1774	
sd	536		580	
Charcuterie (times/w)				
Mean	1·6		1·5	
sd	2·1		2·1	
Meat (times/w)				
Mean	4·6		4·7	
sd	2·4		2·3	
Alcohol (times/w)				
Mean	13·1		13·2	
sd	16·3		17·0	
Total DP*,|| (frequency of consumption)				
Low	76	22·2	40	20·7
Moderate	186	54·2	104	53·9
High	81	23·6	49	25·4
Milk*,¶ (frequency of consumption)				
Low	86	25·1	43	22·3
Moderate	173	50·4	91	47·1
High	84	24·5	59	30·6
Fresh DP** (frequency of consumption)				
Low	86	25·1	49	25·1
Moderate	163	47·5	100	51·3
High	94	27·4	46	23·6
Cheese†† (frequency of consumption)				
Low	75	21·9	38	19·5
Moderate	180	52·5	108	55·4
High	88	25·7	49	25·1
	Mean	sd	Mean	sd
Brain structure‡‡				
GMV (cm^3^)	463·1	42·4	445·8	41·1
MTLV (cm^3^)	15·3	1·9	15·2	2·1
TIV (cm^3^)	906·3	91·1	915·6	91·9
Mean CT in AD-vulnerable regions (mm)	3·1	0·1	3·1	0·1

DP, dairy products; GMV grey matter volume; MTLV, medial temporal lobe volume; TIV, total intracranial volume; CT, cortical thickness; AD, Alzheimer’s disease.

Values are *n* (%) except where mentioned.

* Missing data for 3-year MRI sample: ApoE4 carrier status (*n* 9), stoutness (*n* 1); for 9-year MRI sample: ApoE4 carrier status (*n* 8), stoutness (*n* 1), frequency of consumption of total DP (*n* 2) and milk (*n* 2).

† MRI.

‡ ε4 allele of the apo E.

§ Based on BMI: underweight < 20 kg/m² for those < 70 years old and < 22 kg/m² for ≥ 70 years old, normal (20–27) kg/m² for < 70 years old and (22–27) kg/m² for ≥ 70 years old, and overweight/obesity > 27 kg/m².

|| Frequency of consumption of total dairy products: low ≤ 2, moderate (2–4) and high ≥ 4 times/d.

¶ Frequency of consumption of milk: low 0, moderate (0–1) and high > 1 times/d.

** Frequency of consumption of fresh dairy products: low < 0·5, moderate (0·5–1·5) and high > 1·5 times/d.

†† Frequency of consumption of cheese: low ≤ 0·5, moderate (0·5–1·5) and high > 1·5 times/d.

‡‡ Brain structure: GMV, MTLV, TIV and mean CT in Alzheimer’s disease (AD)-vulnerable regions^(25)^.

Among the 343 participants who underwent the 3-year MRI exam, the mean age was 74·1 (sd, 3·8) years at baseline, and 60·6 % were female. The 195 participants who underwent the 9-year MRI exam were slightly younger at baseline (73·4 (sd 3·7) years), with the same proportion of women (60·0 %).

In both samples, a low frequency of T-DP consumption was defined as the consumption of T-DP ≤ 2 times per d (22·2 % and 20·7 % of those who underwent the 3-year MRI exam and the 9-year MRI exam, respectively), a moderate frequency of T-DP consumption was defined as the consumption of T-DP 2–4 times per d (54·2 % and 53·9 % according to the MRI exam period, respectively) and a high frequency of consumption was defined as the consumption of T-DP ≥ 4 times per d (23·6 % and 25·4 % according to the MRI exam period, respectively). With regard to the DP subtypes, the lowest frequencies of consumption were defined as no milk intake, the consumption of F-DP < 0·5 times per d and the consumption of cheese ≤ 0·5 times per d; the moderate frequencies were defined as the consumption of milk 0–1 times per d, the consumption of F-DP 0·5–1·5 times per d and the consumption of cheese 0·5–1·5 times per d. The highest frequencies of consumption were defined as the consumption of milk > 1 time per d and the consumption of F-DP or cheese > 1·5 times per d.

### Association between frequency of consumption of total dairy products and dairy products subtypes and brain structure

After adjusting for covariates, the frequencies of consumption of T-DP or DP subtypes (i.e. milk, F-DP and cheese) were not significantly associated with structural brain measures at the 3-year MRI exam (Table 2).

**Table 2.** Associations of frequency of consumption of total dairy products, milk, fresh dairy products and cheese with brain structure assessed 3 years later, Bordeaux sample of the Three-City study, 2001–2006, *n* 343

	Model 1			Model 2			Model 3		
	*β* *	95 % CI	*P* **	*β* *	95 % CI	*P* **	*β* *	95 % CI	*P* **
GMV^a^ (in cm^3^)									
Frequency of consumption									
Total DP†									
Low	1			1			1		
Moderate	0·72	–3·80, 5·24	0·80	0·01	–4·45, 4·45	0·99	0·06	–4·42, 4·54	0·98
High	–0·63	–5·97, 4·71	0·82	–1·77	–7·03, 3·50	0·86	–1·20	–6·64, 4·25	0·76
Milk‡									
Low	1			1			1		
Moderate	2·57	–1·87, 7·01	0·80	1·89	–2·52, 6·29	0·96	2·01	–2·59, 6·60	0·98
High	1·92	–3·31, 7·15	0·82	0·46	–4·73, 5·64	0·86	0·83	–4·56, 6·22	0·76
Fresh DP§									
Low	1			1			1		
Moderate	–1·25	–5·70, 3·21	0·80	–0·73	–5·10, 3·65	0·99	–0·70	–5·09, 3·69	0·98
High	0·60	–4·52, 5·72	0·82	0·69	–4·32, 5·70	0·86	0·94	–4·17, 6·06	0·76
Cheese||									
Low	1			1			1		
Moderate	–0·60	–5·32, 4·13	0·80	–1·69	–6·37, 3·00	0·96	–1·55	–6·28, 3·17	0·98
High	–2·31	–7·63, 3·00	0·82	–3·22	–8·51, 2·07	0·86	–2·67	–8·06, 2·71	0·76
MTLV^b^ (in cm^3^)									
Frequency of consumption									
Total DP†									
Low	1			1			1		
Moderate	–0·35	–0·74, 0·04	0·30	–0·37	–0·76, 0·02	0·26	–0·37	–0·77, 0·02	0·24
High	–0·44	–0·90, 0·02	0·17	–0·47	–0·93, −0·01	0·16	–0·49	–0·97, −0·01	0·16
Milk‡									
Low	1			1			1		
Moderate	–0·11	–0·49, 0·28	0·74	–0·13	–0·52, 0·26	0·68	–0·16	–0·56, 0·25	0·67
High	–0·25	–0·70, 0·21	0·30	–0·29	–0·74, 0·17	0·29	–0·32	–0·79, 0·16	0·25
Fresh DP§									
Low	1			1			1		
Moderate	–0·12	–0·51, 0·26	0·74	–0·13	–0·52, 0·25	0·68	–0·13	–0·52, 0·25	0·67
High	–0·39	–0·83, 0·06	0·17	–0·39	–0·83, 0·05	0·16	–0·41	–0·86, 0·04	0·16
Cheese||									
Low	1			1			1		
Moderate	–0·07	–0·48, 0·34	0·74	–0·07	–0·48, 0·34	0·74	–0·08	–0·50, 0·34	0·70
High	–0·24	–0·70, 0·22	0·30	–0·23	–0·69, 0·24	0·34	–0·25	–0·72, 0·23	0·31
Mean CT in AD-vulnerable regions^c^ (in mm)									
Frequency of consumption									
Total DP†									
Low	1			1			1		
Moderate	–0·03	–0·07, 0·01	0·27	–0·04	–0·08, −0·01	0·12	–0·04	–0·08, −0·03	0·11
High	–0·03	–0·07, 0·02	0·46	–0·03	–0·08, 0·01	0·44	–0·04	–0·08, 0·01	0·40
Milk‡									
Low	1			1			1		
Moderate	–0·01	–0·04, 0·03	0·91	–0·01	–0·04, 0·03	0·63	–0·01	–0·05, 0·03	0·62
High	0·01	–0·03, 0·05	0·95	–0·01	–0·05, 0·04	0·87	–0·01	–0·05, 0·04	0·89
Fresh DP§									
Low	1			1			1		
Moderate	–0·03	–0·06, 0·01	0·27	–0·02	–0·06, 0·01	0·39	–0·02	–0·06, 0·01	0·38
High	–0·03	–0·07, 0·01	0·46	–0·02	–0·06, 0·02	0·50	–0·02	–0·06, 0·02	0·56
Cheese||									
Low	1			1			1		
Moderate	0·01	–0·02, 0·05	0·63	0·01	–0·03, 0·05	0·63	0·01	–0·03, 0·05	0·62
High	–0·01	–0·04, 0·04	0·98	–0·01	–0·05, 0·04	0·87	–0·01	–0·05, 0·04	0·89

GMV, grey matter volume; DP, dairy products; MTLV, medial temporal lobe volume; CT, cortical thickness; AD, Alzheimer’s disease.

* *β* coefficients estimated by linear regression adjusted for age, sex, education level, ε4 allele of the apo E, total intracranial volume in model 1, additionally for diabetes, stroke hospitalisation, number of medications taken per d, stoutness in model 2, and additionally for total energy intake, frequencies of consumption of charcuterie, meat and alcohol in model 3. For each DP subtype, each exposure was mutually adjusted for the two others.

** Adjusted *P*-value using the Benjamini–Hochberg method for multiple testing corrections.

† Frequency of consumption of total dairy products: low ≤ 2, moderate (2–4) and high ≥ 4 times/d.

‡ Frequency of consumption of milk: low 0, moderate (0–1) and high > 1 times/d.

§ Frequency of consumption of fresh dairy products: low < 0·5, moderate (0·5–1·5) and high > 1·5 times/d.

|| Frequency of consumption of cheese: low ≤ 0·5, moderate (0·5–1·5) and high > 1·5 times/d.

At the time of the 9-year MRI exam, there was no significant association between the moderate and highest frequencies of consumption of T-DP, milk or cheese and MTLV (Table 3). However, compared with the lowest frequency of F-DP intake (< 0·5 times/d), the highest frequency intake (> 1·5 times/d) was significantly associated with a lower MTLV (*β* = −1·09 cm^3^, 95 % CI − 1·83, −0·36 cm^3^, corrected-*P* = 0·02, in the model adjusted for age, sex, education level, ApoE4 carrier status, total intracranial volume and the frequency consumption of milk and cheese), while no significant association was observed for the moderate frequency intake of F-DP after correcting for multiple testing (*β* = −0·67 cm^3^, 95 % CI − 1·30, −0·04 cm^3^, corrected-*P* = 0·15) (Table 3, model 1). The inclusion of additional covariates considered as potential confounders (diabetes, a history of stroke, the number of medications taken per d and stoutness in model 2; total energy intake and frequencies of consumption of charcuterie, meat and alcohol in model 3) (Table 3) did not meaningfully modify these results. There was no significant association between the frequency consumption of T-DP or the three DP subtypes and GMV or CT in AD-vulnerable regions (Table 3). None of these associations were modulated by sex (P_for interaction_ = 0·57).

**Table 3.** Associations of frequency of consumption of total dairy products, milk, fresh dairy products and cheese with brain structure assessed 9 years later, Bordeaux sample of the Three-City study, 2001–2011, *n* 195

	Model 1			Model 2			Model 3		
	*β* *	95 % CI	*P* **	*β* *	95 % CI	*P* **	*β* *	95 % CI	*P* **
GMV (in mm^3^)									
Frequency of consumption									
Total DP†									
Low	1			1			1		
Moderate	2·53	–4·49, 9·56	0·64	3·19	–3·92, 10·31	0·62	2·95	–4·17, 10·08	0·44
High	–0·47	–8·61, 7·67	0·96	0·05	–8·18, 8·28	0·99	1·02	–7·31, 9·36	0·81
Milk‡									
Low	1			1			1		
Moderate	–3·45	–10·59, 3·69	0·64	–3·46	–10·82, 3·91	0·62	–5·46	–12·89, 1·97	0·44
High	–1·01	–8·67, 6·66	0·96	–1·21	–9·13, 6·71	0·99	–1·52	–9·46, 6·41	0·81
Fresh DP§									
Low	1			1			1		
Moderate	–2·69	–9·48, 4·11	0·64	–2·60	–9·55, 4·36	0·62	–3·32	–10·23, 3·58	0·44
High	0·17	–7·78, 8·13	0·96	0·62	–7·46, 8·70	0·99	1·58	–6·40, 9·56	0·81
Cheese||									
Low	1			1			1		
Moderate	0·52	–6·79, 7·84	0·89	0·85	–6·57, 8·26	0·82	2·91	–4·51, 10·34	0·44
High	–5·89	–14·33, 2·55	0·68	–5·48	–14·04, 3·08	0·83	–4·01	–12·57, 4·55	0·81
MTLV (in mm^3^)									
Frequency of consumption									
Total DP†									
Low	1			1			1		
Moderate	–0·22	–0·87, 0·44	0·93	–0·11	–0·76, 0·53	0·92	–0·10	–0·74, 0·56	0·92
High	–0·35	–1·11, 0·41	0·73	–0·31	–1·06, 0·43	0·81	–0·26	–1·02, 0·50	0·97
Milk‡									
Low	1			1			1		
Moderate	0·03	–0·63, 0·69	0·93	–0·05	–0·71, 0·61	0·92	–0·07	–0·75, 0·61	0·92
High	0·13	–0·58, 0·84	0·96	0·02	–0·68, 0·73	0·95	–0·02	–0·71, 0·74	0·97
Fresh DP§									
Low	1			1			1		
Moderate	–0·67	–1·30, −0·04	0·15	–0·63	–1·25, −0·01	0·19	–0·60	–1·23, 0·03	0·24
High	–1·09	–1·83, −0·36	0·02	–0·99	–1·71, −0·26	0·03	–0·99	–1·72, −0·27	0·03
Cheese||									
Low	1			1			1		
Moderate	–0·09	–0·76, 0·59	0·93	0·03	–0·63, 0·70	0·92	0·04	–0·64, 0·71	0·92
High	–0·02	–0·80, 0·76	0·96	0·09	–0·68, 0·85	0·95	0·10	–0·68, 0·89	0·97
Mean CT in AD-vulnerable regions (in mm)									
Frequency of consumption									
Total DP†									
Low	1			1			1		
Moderate	–0·03	–0·09, 0·03	0·53	–0·02	–0·08, 0·04	0·60	–0·03	–0·08, 0·03	0·45
High	–0·04	–0·11, 0·02	0·36	–0·04	–0·10, 0·03	0·54	–0·03	–0·10, 0·03	0·64
Milk‡									
Low	1			1			1		
Moderate	–0·02	–0·08, 0·03	0·53	–0·02	–0·08, 0·04	0·60	–0·04	–0·10, 0·02	0·45
High	0·01	–0·05, 0·07	0·79	0·01	–0·06, 0·07	0·94	–0·01	–0·06, 0·06	0·97
Fresh DP§									
Low	1			1			1		
Moderate	–0·03	–0·09, 0·03	0·53	–0·03	–0·08, 0·03	0·60	–0·04	–0·09, 0·02	0·45
High	–0·04	–0·10, 0·03	0·38	–0·03	–0·09, 0·04	0·54	–0·02	–0·09, 0·04	0·65
Cheese||									
Low	1			1			1		
Moderate	–0·01	–0·07, 0·05	0·76	0·01	–0·06, 0·06	0·99	0·02	–0·04, 0·08	0·57
High	–0·06	–0·13, 0·01	0·31	–0·05	–0·12, 0·02	0·54	–0·04	–0·11, 0·03	0·64

GMV, grey matter volume; DP, dairy products; MTLV, medial temporal lobe volume; CT, cortical thickness; AD, Alzheimer’s disease.

* *β* coefficients estimated by linear regression adjusted for age, sex, education level, ε4 allele of the apo E, total intracranial volume in model 1, additionally for diabetes, stroke hospitalisation, number of medications taken per d, stoutness in model 2, and additionally for total energy intake, frequencies of consumption of charcuterie, meat and alcohol in model 3. For each DP subtype, each exposure was mutually adjusted for the two others.

** Adjusted *P*-value using the Benjamini–Hochberg method for multiple testing corrections.

† Frequency of consumption of total dairy products: low ≤ 2, moderate (2–4) and high ≥ 4 times/d.

‡ Frequency of consumption of milk: low 0, moderate (0–1) and high > 1 times/d.

§ Frequency of consumption of fresh dairy products: low < 0·5, moderate (0·5–1·5) and high > 1·5 times/d.

|| Frequency of consumption of cheese: low ≤ 0·5, moderate (0·5–1·5) and high > 1·5 times/d.

As often performed in brain imaging studies, to help interpret the scale of brain volume alterations, we compared the effect estimate for the highest frequency of consumption of F-DP with the estimated reduced MTLV expected with advancing age in multivariable model 1. Each additional 1-year increase was associated with a significant decrease of 0·18 cm^3^ (95 % CI − 0·25, −0·11 cm^3^) in the MTLV. Thus, the effect estimate for consuming F-DP > 1·5 times/d was equivalent to a 6·1-year delay in MTLV loss.

## Discussion

In this study of older adults without dementia, we found no association between the frequency of T-DP, milk or cheese intake and brain structure evaluated by MRI 3 or 9 years after the completion of the dietary survey. However, participants with the highest frequency of consumption of F-DP (> 1·5 times/d), evaluated in 2001–2002, had a significantly lower MTLV compared with those with the lowest frequency of consumption of F-DP (< 0·5 times/d), 9 years after the completion of the dietary survey. Compared with individuals exhibiting the lowest frequency of consumption of F-DP, in those with the highest frequency, the estimated reduction in the MTLV was equivalent to the effect estimate of a 6-year increase in age.

The available literature on DP intake and cognitive ageing has mainly focused on clinical outcomes (i.e. cognitive decline or dementia risk) with mixed results overall^(11–14)^, and no studies have investigated F-DP intake alone. Studies on the specific association of DP, with or without DP subtypes and with brain structures assessed with MRI, providing relevant objective measures are scarce. To our knowledge, five previous cross-sectional studies^(17–21)^ investigated the association between components of the MeDi, including DP, and imaging biomarkers in samples of a few hundred individuals, cognitively healthy individuals^(18–20)^ or individuals without dementia^(17,21)^. Similar to our results, no association was observed between the T-DP intake and brain structure, including the GMV^(17,20)^, total white matter volume^(17,20)^, volumes of specific regions of interest^(17–20)^ or CT in AD-vulnerable regions^(17,18)^, in most of these studies. Finally, when focusing on food groups including in the MeDi among 146 individuals without dementia from the 3C Bordeaux cohort, we reported that a higher intake of T-DP was associated with decreased white matter integrity in regions limited to the body and genu of the corpus callosum 9 years after dietary assessment^(21)^.

To date, no study has distinguished the intake of milk, F-DP and cheese, limiting the possibility of comparing the specific association reported in the present study between F-DP intake and MTLV with previous reports.

We hypothesised that higher DP consumption could preserve brain structures. Indeed, several nutrients provided in part by DP intake have been presumed to have neuroprotective, antioxidant and anti-inflammatory properties^(9,10)^. For instance, Camfield *et al.* suggested that several specific components of DP, notably from fermented products, including bioactive peptides, colostrinin, proline-rich polypeptides, *α*-lactalbumin, Ca and probiotics, might promote healthy brain function during ageing^(9)^. Several mechanisms are in accordance with this hypothesis. First, glutathione is an antioxidant that is associated with neurodegeneration if provided in low quantities. Interestingly, greater DP consumption in older adults has been associated with higher cerebral glutathione concentrations^(10)^. Moreover, in adults and aged mice, Trp-Tyr peptides, derived from digested fermented DP, have been shown to improve memory function^(36)^. Indeed, the production of oleamide and dehydroergosterol from fermented DP in mice is responsible for reduced microglial inflammatory responses and neurotoxicity^(37,38)^. Among other nutrients of interest, the fat content of F-DP is unlikely to fully explain our results, as the frequency of cheese consumption (with larger fat content) was not related to brain structural outcomes in the present analysis. Interestingly, the highest consumers of F-DP in the 3C cohort were also those who consumed less *n*-3 PUFA^(22)^. Thomas *et al.* recently reported an association between higher levels of plasma *n*-3 PUFA and a reduced MTLV, a lower risk of dementia and less cognitive decline in the 3C cohort^(27)^. Finally, individuals in the present analysis with the highest frequency of consumption of F-DP also exhibited a lower intake of vitamin B_12_, a vitamin with putative benefits for cognitive performance^(39)^, than those with the lowest frequency of consumption of F-DP^(22)^. Notably, after adjustment for dietary *n*-3 and B_12_, our results remained unchanged. Despite convincing biological plausibility, our present findings did not confirm our initial hypothesis or the mechanisms involved.

The strengths of our population-based study included comprehensive evaluation of DP subtypes. Moreover, our analyses were controlled for a large set of potential confounders, including sociodemographic and clinical characteristics and the intake of some foods.

The present study also had a few limitations. The single assessment of DP consumption, limited to the frequency of consumption, might have induced misclassification at baseline and prevented us from assessing portion sizes. While dietary diversity changes affect cognitive function^(40)^, we assumed that dietary habits did not change during the follow-up, which cannot be determined since the subsequent FFQ administered did not detail DP by subtype. However, we already reported that the frequency of consumption of major food groups seemed relatively stable in the 3C Bordeaux cohort during follow-up^(21)^. Another limitation is the design of the study with a gap between the exposures and the outcomes. Although two MRI exams were carried out 3 and 9 years after the completion of the dietary assessment, the change in the MRI scanner between the two assessments did not allow for the longitudinal analysis of brain atrophy or cortical thinning over time. In addition, the associations only observed after 9 years, and not 3 years, could be explained by measurement sensitivity, with the increase in performance between the two MRI scanners. Additionally, the imaging ancillary study included healthier (selected without exclusion for MRI contraindications) and younger participants than the overall cohort population due to technical inclusion criteria and acceptance-related selection. Notably, as the MRI examinations were part of a complementary ancillary study, financial and logistical constraints unfortunately did not allow us to perform these tests on the initial sample. Finally, the exclusion of individuals with prevalent dementia induces a selection bias, which is nevertheless quite small in view of the number of excluded individuals (*n* 1 for the 3-year MRI and *n* 6 for the 6-year MRI). However, if we had chosen to include these individuals in the study, we would not have been able to have absolute confidence in the dietary data collected.

In conclusion, in this population-based cohort of French older adults, there was no association between the frequency of T-DP, milk or cheese intake and brain structures, while the consumption of F-DP more often than 1·5 times/d was significantly associated with a lower MTLV, a validated marker of AD, 9 years after the completion of the dietary survey. This original study should be replicated in different settings before definite conclusions can be drawn.

## Supplementary material

For supplementary material accompanying this paper visit https://doi.org/10.1017/S0007114523001551. click here to view supplementary material
