# Supplementary material for: Dairy products and brain structure in French older adults
Source: Br J Nutr. 2023 Sep 11;131(3):512–20. doi: 10.1017/S0007114523001551 (PMC10784124; doi:10.1017/S0007114523001551)
Supplement: Supplementary file 1 [file S0007114523001551sup001.docx]

**Supplementary Figure Methods**

**Flow chart of participants included in the study, the Three-City Bordeaux study, 2001-2011**

**
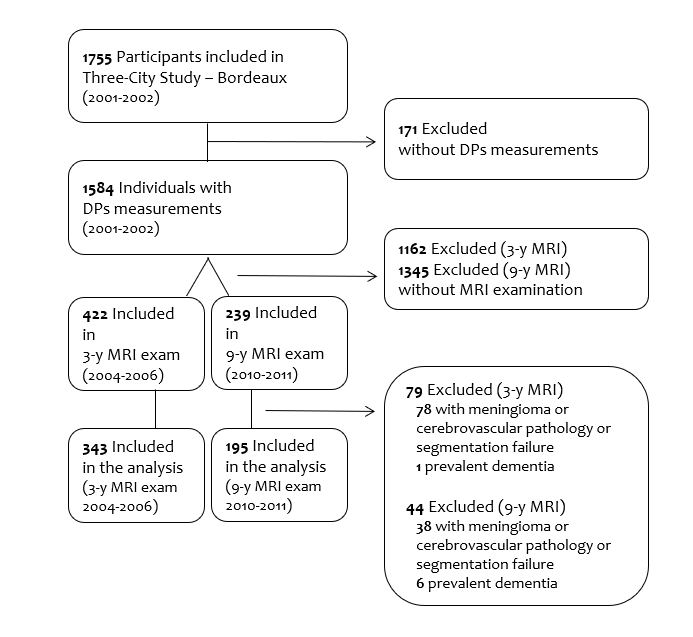
**

**Supplementary Methods**

**Assessment of brain structure**

MRI acquisition

In 2004-2006, the first MRI examination was performed on a 1.5-T Gyroscan Interra system (Philips Medical System, The Netherlands) equipped with a quadrature head coil. Anatomical high resolution MRI volumes were acquired in transverse plane using a T1 (3D magnetization prepared rapid gradient echo [MPRAGE]) weighted sequence, with the following parameters: repetition time [TR]/echo time [TE] 8.5/3.9 ms, flip angle 10°, matrix size 256 x256, field of view [FOV] 240 mm, yielding 124 slices and slice thickness of 1 mm, voxel size 0.94x0.94x1 mm^3^.

In 2010-2011, the second MRI exam was performed using an ACHIEVA 3T scanner (Philips Medical System, The Netherlands) equipped with a SENSE 8-channel head coil. Anatomical high resolution MRI volumes were acquired in transverse plane using a three-dimensional MPRAGE weighted-T1 sequence with the following parameters: TR/TE=8.2/3.5 ms, 7-degree flip angle, FOV 256×256 mm^2^ to cover the whole brain, yielding 180 contiguous slices, voxel size 1×1×1 mm^3^.

Head motions were minimized by the use of tightly padded clamps attached to the head coil. All acquisitions were aligned on the anterior commissure-posterior commissure plane.

MRI processing

Each subject's anatomical images were processed using cortical segmentation of Freesurfer (v5.1, http://surfer.nmr.mgh.harvard.edu) with the Destrieux parcellation atlas [1]. Hippocampal and amygdalar parcellations were done using fMRIB's Integrated Registration and Segmentation Tool [2], part of FSL [3]. Results of the segmentation were checked for global accuracy of the anatomical delineation: poor segmentation quality was identified based on the description of grey matter and hippocampal volumes and discarded if needed after visual inspection by three trained-operators.

**References**

[1] Destrieux C, Fischl B, Dale A, Halgren E. Automatic parcellation of human cortical gyri and sulci using standard anatomical nomenclature. NeuroImage 2010;53:1–15. <https://doi.org/10.1016/j.neuroimage.2010.06.010>.

[2] Patenaude B, Smith SM, Kennedy DN, Jenkinson M. A Bayesian model of shape and appearance for subcortical brain segmentation. NeuroImage 2011;56:907–22. <https://doi.org/10.1016/j.neuroimage.2011.02.046>.

[3] Smith SM, Jenkinson M, Woolrich MW, Beckmann CF, Behrens TEJ, Johansen-Berg H, et al. Advances in functional and structural MR image analysis and implementation as FSL. NeuroImage 2004;23 Suppl 1:S208-219. https://doi.org/10.1016/j.neuroimage.2004.07.051.
